# Impact of 12-Week Moderate-Intensity Aerobic Training on Inflammasome Complex Activation in Elderly Women

**DOI:** 10.3389/fphys.2022.792859

**Published:** 2022-02-22

**Authors:** Marta Gomarasca, Katarzyna Micielska, Martina Faraldi, Marta Flis, Silvia Perego, Giuseppe Banfi, Ewa Ziemann, Giovanni Lombardi

**Affiliations:** ^1^Laboratory of Experimental Biochemistry and Molecular Biology, IRCCS Istituto Ortopedico Galeazzi, Milan, Italy; ^2^Department of Physical Education and Lifelong Sports, Poznań University of Physical Education, Poznań, Poland; ^3^Doctoral School, Gdańsk University of Physical Education and Sport, Gdańsk, Poland; ^4^Department of Physiology, Gdańsk University of Physical Education and Sport, Gdańsk, Poland; ^5^Vita-Salute San Raffaele University, Milan, Italy; ^6^Department of Athletics, Strength and Conditioning, Poznań University of Physical Education, Poznań, Poland

**Keywords:** aging, NLRP3 inflammasome, pro-inflammatory cytokines, physical activity, aerobic exercise

## Abstract

Aging often associates with a chronic low-grade inflammatory status that can be consequent to the activation of Toll-like receptors (TLRs) and the downstream NLR family pyrin domain containing 3 (NLRP3) inflammasome and causes a chronic secretion of pro-inflammatory cytokines. Since exercise has known anti-inflammatory effects, we investigated the effect of Nordic walking training on inflammasome activation and downstream effectors in elderly women. A population of elderly women was divided into EXP (*n* = 29) that completed 12 weeks of the moderate-intensity aerobic training program and CTRL (*n* = 29), performing no activity. Blood samples were taken before and after the first (T1-pre and T1-post, respectively) and last (T2-pre and T2-post, respectively) exercise unit. Inflammasome activation status was assessed by whole blood NLRP3 and TLR4 expression by RT-qPCR. Serum levels of IL-1β, IL-6, TNFα, and IL-18 cytokines were assayed by multiplex fluorescent beads-based immunoassays or ELISA. NLRP3 and TLR4 levels were reduced 2 folds between T1-pre and T2-pre and induced at T2-post, compared to T2-pre, by 2.6- and 2.9-fold, respectively. A single exercise bout elicited a 1. 38-, 1. 5-, and 1.36-fold rise of IL-1β, TNFα, and IL-6 concentration, respectively, although not significant, at the beginning of the training (T1-pre vs. T1-post), a 1.4-fold decrease for IL-1β and TNFα at the end of the training (T1-pre vs. T2-pre), and a 2-, 1.8- and 1.26-fold increase after the last exercise session (T2-pre vs. T2-post) for the three cytokines. When stratifying the population based on BMI in normal weight (NW) and overweight (OW), NLRP3 and TLR4 expression was affected only in NW. As for inflammatory cytokines, IL-1β was modulated in NW at the beginning of the training, whereas in OW at the end of the training; for TNFα, this time-dependent modulation was significant only in OW. Applied aerobic training affected the resting expression of inflammasome constituents (NLRP3 and TLR4) and levels of downstream effectors (IL-1β, TNFα, and IL-6). However, at the end of the program, participants acquire an acute inflammatory response to exercise that was absent at baseline. Future studies would have to define the molecular mechanisms associated with, and how to potentiate, the exercise-associated inflammatory response.

## Introduction

Aging is associated with several biological changes that profoundly affect cell and tissue functions and hesitates in the increased risk of developing diseases, frailty, injuries, disability, hospitalization, and, consequently, mortality. Among the plethora of altered functions, aging associates with a persistent state of chronic low-grade inflammation (LGI), also referred to as inflammaging (Franceschi et al., 2000). LGI is, in turn, associated with onset and development of most diseases including metabolic dysfunctions (e.g., obesity, impaired glucose tolerance, metabolic syndrome, and type 2 diabetes), muscle-skeletal failure (e.g., osteopenia, osteoporosis, and sarcopenia), cancers, neurodegenerative diseases, and aging itself (Franceschi et al., 2007).

Inflammation is a complex homeostatic response to harmful stimuli that protects the organism and promotes tissue repair and regeneration after injury, by orchestrating the innate immune response (Franceschi et al., 2007). The innate immune response is primed by the activation of pattern-recognition receptors (PRRs), as the Toll-like receptors (TLRs), C-type lectin receptors (CLRs), retinoic acid-inducible gene (RIG)-I-like receptors (RLRs), and NOD-like receptors (NLRs), by pathogen-associated molecular patterns (PAMPs). Injured cells can also activate the innate inflammatory response through the release of endogenous damage-associated molecular patterns (DAMPs), in the absence of pathogens (Gomarasca et al., 2020). During aging, alterations in T cell function, immune-senescence, extracellular matrix alterations, unfavorable changes in body composition (increased fat mass), and foci of chronic infections feed the presence of DAMPs and PAMPs into the circulation and keep the inflammatory response chronically activated (Franceschi et al., 2007; Mejias et al., 2018). This condition, known exactly as the LGI, is characterized by slightly and chronically increased plasma levels of pro-inflammatory cytokines, such as interleukin (IL)-6, IL-1β, tumor necrosis factor (TNF)α, and C-reactive protein (CRP). This pro-inflammatory state is caused by the activation of TLRs, upon PAMPs and DAMPs recognition, and the downstream signaling that culminates in the activation of NF-κB and MAPK pathways that promotes the expression of pro-inflammatory cytokines (Kawasaki and Kawai, 2014).

A key modulator of age-related systemic LGI is the NLR family pyrin domain containing 3 (NLRP3) inflammasome. Inflammasomes are multimeric protein complexes that assemble in the cytosol after sensing both PAMPs and DAMPs. Inflammasomes are involved in the activation of caspase-1 that in turn processes the pro-inflammatory cytokines IL-1β and IL-18 into their bioactive mature forms. The NLRP3 inflammasome needs to be primed by the activation of the TLR4 that, throughout NF-κB signaling, leads to the increased expression of the NLRP3 protein (Guo et al., 2015). Among others, aging is associated with elevation of intracellular and extracellular levels of uric acid, reactive oxygen species (ROS), free fatty acids (fFAs), ceramides, free cholesterol, oxidized low-density lipoproteins (LDL), advanced glycation end products, as well as with the alteration of the microbial community and the consequent increase in the level of microbial-derived constituents in blood. All these age-associated danger signals, contribute to the activation of the NLRP3 inflammasome and the subsequent triggering of the so-called age-related inflammation (or inflammaging) (Gritsenko et al., 2020). Thus, the NLRP3 inflammasome is a major sensor of age-related accumulation of DAMPs, in absence of apparent infection (Youm et al., 2013).

Powerful and effective regulators of chronic LGI are physical exercise and training. Both aerobic and resistance chronic exercises exert beneficial effects on the modulation of the inflammatory response (Gerosa-Neto et al., 2020; Padilha et al., 2021), reduction of chronic LGI in the elderly population (Dalle et al., 2017; Duggal et al., 2019), as well as, on the improvement of the metabolic profile (Lira et al., 2017; Da Silva et al., 2020). Exercise, which is defined as a planned, structured, and repeated physical activity (PA), can reduce not only the baseline inflammatory status of chronic LGI but also the acute inflammatory response against harmful stimuli (Beyer et al., 2012). However, only a few studies investigated the effects of PA on inflammasome activation and none has focused on Nordic walking. A resistance training (RT) program in healthy elderly reduced the protein expression of TLR4 and its downstream signaling effectors, leading to an overall improvement of the inflammatory status (Rodriguez-Miguelez et al., 2014). Similarly, another study related to RT intervention in elderly demonstrated the downregulation of NLRP3 protein and a decreased caspase-1-to-pro-caspase-1 ratio in peripheral blood mononuclear cells (PBMCs), highlighting the possible beneficial effect of RT in limiting the inflammatory reactivity (Mejias-Pena et al., 2017). A recent study has demonstrated that also moderate-intensity chronic aerobic exercise may reduce TLR4 and NLRP3 mRNA expression in PBMCs and circulating levels of IL-1β and IL-18 in young males (Khakroo Abkenar et al., 2019). Moreover, it was revealed that 12 weeks of moderate-intensity aerobic training combined with RT brought cardiometabolic benefits in adults with metabolic syndrome (Da Silva et al., 2020). Previously published papers indicated that Nordic walking training induces a reduction in circulating levels of the autophagy protein high mobility group box 1 (HMGB1) in elderly women and an increase the myokine irisin (Gmiat et al., 2017). Thereby, moderate-intensity aerobic training, as Nordic walking, seems to positively affect the inflammatory status and it happens mainly throughout the modulation of the innate immunity function (Padilha et al., 2021), whose activation mainly relay on TLR4 signaling and the consequent inflammasome activation, especially in the elderly subjects that experience an age-associated deregulation of the TLR4-associated inflammatory pathways (Kawasaki and Kawai, 2014; Mejias et al., 2018). Nordic walking, also known as “Scandinavian walking with poles,” is a popular outdoor activity based on specially designed poles for the purpose of activating the upper body during walking. It combines and stimulates skiing, sport walking, and trekking skills: by activating the upper body muscles, the use of poles may increase the length of each step, finally resulting in a faster gait and improved metabolism. Elderly are the most enthusiastic performers maybe because of its open-air, nature friendly, and social behaviors but also because perceived exertion and joint overload are limited thanks to the use of poles, despite the higher heart rate and oxygen consumption compared to a standard walk (Skorkowska-Telichowska et al., 2016). Interestingly, although the moderate intensity, Nordic walking is emerging as a powerful and effective strategy to counteract frailty and, particularly, metabolic- (Muollo et al., 2019) and mobility-associated aspects of frailty, as increased risk of fracture and skeletal muscle wasting (Ossowski et al., 2016; Xu et al., 2016). Within this context, this study aims to unravel the effect of a 12-week Nordic walking moderate aerobic training program on the expression of the main components of inflammasome complex (TLR4 and NLRP3) in whole blood and the downstream cytokine effectors (IL-1β, IL-18, TNFα, and IL-6) in elderly women.

## Materials and Methods

### Study Design

In this intervention case-control study, 70 elderly women (age = 68 ± 8 years old), with a sedentary behavior [according to the American College of Sports Medicine guidelines (ACSM, 2010)] were recruited among church communities, senior citizens’ clubs, and universities of the third age. Participants were randomly assigned to the experimental (EXP, *n* = 35) group, engaged in a 12-week Nordic walking training program, and control (CTRL, *n* = 35) group, not involved in any activity. Recruitment and testing of EXP and CTRL subjects took place at the same period. At enrolment, all subjects underwent a medical examination and were asked to provide information regarding prescribed medications. Exclusion criteria were: uncontrolled hypertension (diastolic blood pressure > 100 mmHg), history of cardiac arrhythmia, cardio-respiratory disorders, and orthopedic problems. Body composition and 2,000 m walking test were determined 1 week prior to the start of the experiment and after 12 weeks of training. Participants belonging to the EXP group were familiarized with the right technique for walking with Nordic walking poles. All subjects were characterized for weight, BMI, and hematological and biochemical markers (as reported in the subsection “Blood Collection and Sample Preparation”).

From the original cohorts, six subjects who either did not attend one blood sampling or did not meet the compliance criteria to training (i.e., <90% participation), were excluded. By the end, 29 subjects were included in the EXP group and 29 in the CTRL group. In order to verify the existence of a BMI-dependent response to the training program, participants were sub-grouped in normal weight (NW; EXP, *n* = 14; CTRL, *n* = 12), with BMI < 25 kg/m^–2^, and overweight/obese (OW; EXP, *n* = 15; CTRL, *n* = 17), with BMI ≥ 25 kg/m^–2^.

The population was not stratified according to Vitamin D intake, since many participants used to take this supplement as a general recommendation from the Ministry of Health for seniors in Poland. The levels of Vitamin D were measured to be in the range of 32–55 ng/mL for the whole population.

The study received the official approval of the Bioethical Committee of the Regional Medical Society in Gdansk (KB-34/18) and was registered as clinical trial with the ID: NCT03417700, in accordance with the Declaration of Helsinki. All participants were given detailed information about experiment, procedures, risks, and benefits of the study and gave their written consent to participation.

### Training Protocol

Participants from EXP group met three times a week (Monday, Wednesday, and Friday), 1 h after eating a light breakfast. In order to avoid the impact of different diets on training response, all the participants were given the same breakfast on the day the tests were collected and were asked not to change their eating habits during the training period. CTRL subjects were also asked to maintain unaltered their lifestyle habits and to keep their PA level below 150 min/week (ACSM, 2010). Both groups were instructed not to perform any additional physical activity during the study period. Each training session lasted 1 h, and consisted of 10-min warm-up, 40-min specific Nordic walking training, and 10-min cool-down. Subjects were equipped with standard Nordic walking poles. The same group of research assistants and coaches checked attendance of participants, supervised all training sessions, and performed the tests. Nordic walking training was performed with 60–70% intensity of the maximal heart rate (HR) obtained during the supervised 2000 m walking test. This test was performed on a flat floor, according to the previously described procedure (Mieszkowski et al., 2018). This test, as well as each training unit, was monitored using Garmin Forerunner 405 with a built-in GPS in order to record distance. The model of Garmin Forerunner 405 was equipped with additional HR sensor. The participants were encouraged to maintain the highest possible pace during the 2,000 m walking test to achieve the highest intensity, but they were not allowed to run. Time was measured using photoelectric cells (Racetime 2 SF, Microgate, Bolzano, Italy) with an accuracy of 0.001 s. The start of the movement was signaled by the instructor. The information about 60–70% HR max intensity achieved during every training session was monitored for each participant individually by coach. To evaluate the maximal oxygen capacity a mathematic formula was applied: VO_2_ max = 116.2-2.98 Time-0.11HR-0.14Age-0.39BMI (Laukkanen et al., 2000; Kortas et al., 2015). The EXP group completed 12 weeks of Nordic walking training, which included 36 training units. During the entire training program, participants in the EXP group covered a total distance of almost 120 km. Only subjects, who attended at least 90% of the total amount of training units, were considered as completing the protocol.

### Blood Collection and Sample Preparation

Blood samples were taken from the antecubital vein by two professional nurses. For the EXP group, blood samples were collected at baseline, before and after the first session of Nordic walking (T1-pre and T1-post, respectively), immediately before the last exercise session, after 12 weeks of training (T2-pre), and immediately after the last training session (T2-post). Post-exercise blood drawings were performed within 15 min from the end of the exercise session. For the CTRL group, blood was sampled only at T1 and T2, corresponding to the T1-pre and T2-pre, respectively, of the EXP cohort. The blood was collected at rest, under fasting condition, between 7:00 and 8:00 a.m. Ethylendiaminotetraacetate dipotassium salt (K2EDTA)-anticoagulated blood (K2EDTA Vacutainer^®^, Becton Dickinson, and Co., Franklin Lakes, NJ, United States) was used for hematological characterizations and RNA extraction. The hematological assessment was performed only at T1-pre and T2-pre for the EXP group and at both T1 and T2 for the CTRL group, and included: hemoglobin [Hb], hematocrit (Ht%), red blood cells (RBC), mean corpuscular volume (MCV), mean corpuscular hemoglobin (MCH), mean corpuscular hemoglobin content (MCHC), red cells distribution width – coefficient of variation (RDW-CV), platelets count (Plt), mean platelet volume (MPV), total white blood cell count (WBC), absolute and relative counts of neutrophils (Neu), lymphocytes (Ly), monocytes (Mo), eosinophils (Eo), and basophils (Ba). Serum was obtained from blood collected into SST II Advance™ tubes with clot activator (Becton Dickinson, and Co.) and was used for the characterization of the metabolic [total cholesterol (TChol), high-density lipoproteins (HDL), low-density lipoproteins (LDL), triglycerides (TG), iron, and ferritin] and inflammatory (as described below) profiles, measured only at T1-pre and T2-pre for the EXP group and at both T1 and T2 for the CTRL group. Samples were centrifuged at 2,000 × *g*, for 10 min, at 4°C and stored at –80°C until later analysis. Guidelines for the correct management of the pre-analytical phase were strikingly followed (Banfi et al., 2010; Dugue et al., 2018; Faraldi et al., 2020). Since [Hb], Ht%, RBC, MCV, MCH, MCHC, RDW-CV, Plt, and MPV remained stable during the observation and did not differ among any of the groups, and are, however, mostly irrelevant to the aim of the current study, they were not further discussed.

Further, 25-hydroxy vitamin D [25-(OH)D], the most reliable marker of vitamin D status (Ferrari et al., 2017), was measured by high-performance liquid chromatography (HPLC) coupled with mass spectrometry (MS) on a Shimadzu LCMS 8050 HPLC system Nexera X2 column with Agilent Eclipse Plus C18 1.8 μm 2.1 × 100 mm columns, according to Gmiat et al. (2017). The measurement was performed at baseline in order to verify that all participants were in a state of sufficiency. Importantly, according to Polish guidelines for seniors, all participants used to be supplemented with vitamin D.

### RNA Extraction

Total RNA was extracted from whole blood using the Direct-Zol miniprep Kits (Zymo Research Co., Orange, CA, United States), following manufacturer instructions. Briefly, three volumes of TRI Reagent^®^ were added to 250 μl of whole blood. After mixing thoroughly and centrifuging at 12,000 × *g* for 30 s at RT, the supernatant was transferred into RNase-free tubes for the subsequent RNA purification. After adding an equal volume of ethanol 95% and mixing, the sample was transferred into the Zymo-Spin™ IC Column and centrifuged at 12,000 × *g*, for 30 s, at RT. Thereafter, the sample was digested with DNase I (6 U/μl) for 15 min at RT. The columns were further washed and the RNA was eluted in 15 μl of DNase/RNase-Free Water by centrifugation at 12,000 × *g* for 30 sat RT. RNA concentration was quantified using a NanoDrop spectrophotometer (Thermo Fisher Scientific, Waltham, MA, United States). RNA purity and integrity were assessed by considering the 260/280 nm and 260/230 nm absorbance ratios, visualized at NanoDrop spectrophotometer and through 1% agarose gel electrophoresis.

### Gene Expression Analysis in Whole-Blood

Total RNA was reverse transcribed using the iScript cDNA Synthesis Kit (Bio-Rad Laboratories, Hercules, CA, United States). RT-qPCR was carried out on a StepOne Plus instrument (Applied Biosystems, Foster City, CA, United States), using TaqMan™ Gene Expression Master Mix and premade 6-Carboxyfluorescein (FAM)-labeled TaqMan assay for *TLR4* (Hs00152939_m1), *NLRP3* (Hs00918082_m1), *PPIB* (Hs00168719_m1), *PGK1* (Hs99999906_m1), *ACTB* (Hs99999903_m1) (Thermo Fisher Scientific). The thermal protocol was as follows: 50°C for 2 min, 95°C for 10 min, followed by 40 amplification cycles at 95°C for 15 s and 60°C for 60 s. Results, reported as quantification cycle (Cq) values, were analyzed by the GenEx software ver. 6 (Exiqon A/S, Vedbaek, Denmark). The relative expression of each gene was calculated by the 2^–ΔΔCq^ method, using PPIB and PGK1 as reference genes. Analysis of target genes was performed on the overall included subjects and on subjects stratified for BMI. Results on gene expression are reported as median (minimum value to maximum value).

### Selection of Reference Genes

PPIB, PGK1, and ACTB were assayed as reference genes. Expression level analysis was performed comparing normalized expression levels calculated as follows:


(1) Δ⁢C⁢q=C⁢q⁢r⁢g-g⁢e⁢o⁢m⁢e⁢a⁢n⁢r⁢g

Cq rg: quantification cycle of a reference gene in a sample.

geomean rg: geometrical mean of the Cq of PPIB, PGK1, and ACTB of all samples.

The heatmap analysis was performed using the tool provided by GenEx software (Exiqon). An in-depth analysis of the normalization strategies was performed as previously described (Faraldi et al., 2019). Expression stability of PPIB, PGK1, and ACTB, was analyzed using the NormFinder (Andersen et al., 2004) and GeNorm (Vandesompele et al., 2002) algorithms provided by the GenEx software.

### Cytokines Analysis

The pro-inflammatory cytokines IL-1β, IL-6, and TNFα were quantified in serum with a multiplex customized Human High Sensitivity Cytokine B Premixed Mag Luminex Performance Assay (R&D Systems, Minneapolis, MN, United States). Samples were analyzed in duplicates and read on a MAGPIX^®^ Multiplex System (Luminex^®^ Co., Austin, TX, United States). The assay sensitivities were 0.146 pg/mL for IL-1β, 0.135 pg/mL for IL-6, and 0.250 pg/mL for TNFα. Values under the last point of the standard curve, but above the blank and the minimum detectable dose (0.03 pg/mL for IL-1β, 0.08 pg/mL for IL-6, and 0.13 pg/mL for TNFα) were derived by the Bio-Plex Manager Software. The intra-assay (CV_i_) and inter-assay (CV_b_) coefficients of variation of each analyte were 1.7 and 11.1% for IL-1β, 2.0 and 11.4% for IL-6, and 1.7 and 11.6% for TNFα, respectively.

IL-18 concentrations were measured in serum by Human Total IL-18 ELISA (R&D Systems), following manufacturer instructions. The assay-specific sensitivity was 5.15 pg/mL. Maximum intra-assay (CV_i_) and inter-assay (CV_b_) coefficients of variation were 3.1% and 8.7%, respectively. Readings were performed at λ = 450 nm subtracted of the corresponding readings at λ = 570 nm on a Victor X3 (PerkinElmer, Waltham, MA, United States). Body mass, body composition, and body mass index (BMI) were determined using a multi-frequency impedance analyzer (In Body_720_, Biospace, South Korea). The measurements were performed twice, 1 week before and after the entire intervention, according to McLester et al. (2020).

### Statistical Analysis

The minimum sample size was determined with G*Power (v3.1.9.7) based on IL-6 serum concentrations as the primary endpoint index, being this cytokine largely described as the prototypic mediator of innate immune response and exercise-dependent metabolic regulation (Chowdhury et al., 2020). For sample size calculation was assumed a two-tailed type I α error of 0.05, a power (1-β error probability) > 0.95, a pre-to-post-intervention difference of 18%, and a standard deviation of ±18.54 pg/mL, according to Gmiat et al. (2018). The total estimated sample size was 22.

Statistical analysis was performed with Prism^®^ v6.01 (GraphPad Software Inc., La Jolla, CA, United States). The D’Agostino-Pearson’s normality test (omnibus K2 test) defined non-parametric distributions for most of the parameters analyzed. Thereby, in the descriptive analysis, data are reported as the median and range (minimum to maximum), while the statistical analysis was conducted with non-parametric tests.

In EXP and CTRL groups, age, height, and Vitamin D were analyzed by non-parametric Mann–Whitney test.

In the EXP group, time-dependent changes were analyzed by non-parametric repeated measures Friedman’s test with Dunn’s multiple comparisons (T1-pre vs. T1-post vs. T2-pre vs. T2-post). Comparison of time-dependent changes between EXP and CTRL group and comparison between NW and OW within and between EXP and CTRL groups were performed by two-way ANOVA with Sidak’s multiple comparison test. Differences were considered statistically significant if *p*-values < 0.05, and only significant data were discussed in the text.

The effect size has been calculated by Kendal W for Friedman’s tests, Cohen‘s eta-squared for two-way ANOVA, and Cohen’s *d* for the *post hoc* tests.

## Results

### Characterization of the Study Cohort

The characterization of EXP subjects before the beginning (T1-pre) and before the last exercise session of the 12-week Nordic walking training (T2-pre), and CTRL subjects over the same period (T1 and T2, respectively) is detailed in Tables 1, 2 and Supplementary Table 1. The characterization of the cohorts at these two time-points allows avoiding the alteration of some hematological markers due to the effect of acute exercise, meaning after a session of training, rather than highlighting the differences due to the chronic exercise.

**TABLE 1:** Anthropometrical characteristic of the study cohort.

	CTRL (*n* = 29)					EXP (*n* = 29)						
	T1		T2		*p*-value T1 vs. T2	T1-pre		T2-pre		*p*-value T1-pre vs. T2-pre	*p*-value T1 CTRL vs. T1-pre EXP	*p*-value T2 CTRL vs. T2-pre EXP
	Median (min-to-max)	*p*-value NW vs. OW	Median (min-to-max)	*p*-value NW vs. OW		Median (min-to-max)	*p*-value NW vs. OW	Median (min-to-max)	*p*-value NW vs. OW			
**Age (years)**	68.0 (60.00–78.0)		/		/	69.0 (60.0–78.0)		/		/	0.568	/
*NW*	67.5 (62.0–76.0)	0.763	/	/	/	70.0 (60.0–78.0)	0.502	/	/	/	0.446	/
*OW*	71.0 (60.0–78.0)		/		/	67.0 (60.0–76.0)		/		/	0.836	/
**Height (m)**	1.63 (1.50–1.74)		/			1.63 (1.53–1.78)		/		/	0.243	/
*NW*	1.64 (1.53–1.74)	0.360	/	/	/	1.65 (1.53–1.78)	0.883	/	/	/	0.962	/
*OW*	1.61 (1.50–1.70)		/		/	1.63 (1.53–1.75)		/		/	0.398	/
**Weight (kg)**	67.80 (54.40–93.00)		67.70 (54.10–93.80)		0.928	67.35 (54.40–94.20)		65.80 (54.80–95.60)		0.460	0.731	0.664
*NW*	63.90 (54.40–73.30)	**0.004**	63.25 (54.10–73.80)	**0.004**	1.000	58.70 (54.40–73.80)	**<0.001**	57.60 (54.80–73.80)	**<0.001**	0.996	0.966	0.971
*OW*	71.80 (58.50–93.00)		72.20 (57.70–93.80)		0.986	68.50 (65.80–94.20)		68.70 (65.10–95.60)		0.258	1.000	1.000
**BMI (kg/m^2^)**	26.00 (20.70–34.60)		25.90 (20.60–34.90)		0.909	25.30 (19.80–33.00)		24.80 (19.60–33.10)		0.995	0.261	0.280
*NW*	23.75 (20.70–24.90)	**<0.001**	23.43 (20.60–25.30)	**<0.001**	1.000	22.60 (19.80–24.70)	**<0.001**	22.20 (19.60–24.70)	**<0.001**	0.941	0.681	0.816
*OW*	27.90 (25.30–34.60)		27.10 (24.90–34.90)		0.992	26.90 (25.20–33.00		26.50 (24.50–33.10)		0.915	0.813	0.771

*Description of weight, BMI, and hematologic markers in the entire study cohort and for the sub-cohorts stratified based on BMI (BMI < 25 kg/m^2^: NW; BMI > 25 kg/m^2^: OW, as determined at recruitment). Data are expressed as median (range) since the non-parametric distribution, as assayed by D’Agostino-Pearson’s test. Comparison of age and height between EXP and CTRL were assessed using Mann–Whitney test. Within-group (EXP and CTRL) time-dependent changes in NW and OW subjects were performed by the means of two-way ANOVA with Sidak’s multiple comparison post hoc test. Statistically significant (p-values < 0.05) differences are indicated in bold. NW, normal weight subjects; OW, overweight subjects; BMI, body mass index.*

**TABLE 2:** White blood cell counts.

	CTRL (*n* = 29)					EXP (*n* = 29)						
	T1		T2		*p*-value T1 vs. T2	T1-pre		T2-pre		*p*-value T1-pre vs. T2-pre	*p*-value T1 CTRL vs. T1-pre EXP	*p*-value T2 CTRL vs. T2-pre EXP
	Median (min-to-max)	*p*-value NW vs. OW	Median (min-to-max)	*p*-value NW vs. OW		Median (min-to-max)	*p*-value NW vs. OW	Median (min-to-max)	*p*-value NW vs. OW			
**WBC (×10^9^/L)**	5.58 (3.93–7.90)		5.74 (4.02–9.21)		0.475	5.73 (3.53–8.89)		5.52 (3.71–10.18)		1.000	0.997	0.821
*NW*	5.83 (5.06–7.90)	0.819	5.69 (4.47–8.80)	1.000	0.999	5.30 (3.95–7.56)	0.641	4.79 (3.79–6.86)	0.232	0.968	0.716	0.577
*OW*	5.53 (3.93–7.41)		5.74 (4.02–9.21)		0.375	6.00 (3.53–8.89)		6.25 (3.71–10.18)		0.977	0.751	0.985
**Neu (%)**	50.80 (38.40–70.10)		50.80 (31.90–66.00)		0.635	52.60 (31.70–77.40)		50.70 (38.10–69.30)		0.323	0.905	0.999
*NW*	49.30 (38.40–65.40)	0.995	50.60 (31.90–66.00)	0.974	0.932	51.40 (31.70–77.40)	0.998	48.50 (41.00–67.20)	0.986	0.747	1.000	1.000
*OW*	53.60 (39.90–70.10)		51.40 (42.20–65.90)		0.977	53.50 (35.90–67.60)		52.00 (38.10–69.30)		0.874	1.000	1.000
**Ly (%)**	35.40 (19.10–48.20)		37.10 (21.80–54.90)		0.296	34.85 (13.20–54.90)		37.95 (20.50–49.20)		0.280	0.989	0.995
*NW*	38.70 (23.10–47.20)	0.964	37.05 (23.80–54.90)	0.957	0.834	37.20 (13.20–54.90)	0.999	39.00 (20.50–49.20)	0.976	0.662	1.000	1.000
*OW*	33.80 (19.10–48.20)		37.10 (21.80–46.30)		0.758	34.10 (25.80–49.10)		36.70 (21.50–47.60)		0.874	1.000	1.000
**Mo (%)**	8.20 (6.10–11.00)		8.20 (5.60–11.00)		0.431	8.60 (5.50–13.30)		8.90 (5.60–13.40)		0.889	0.462	0.277
*NW*	8.05 (6.90–10.70)	1.000	7.70 (5.60–10.90)	0.999	0.711	8.50 (6.20–12.00)	1.000	8.70 (5.70–13.40)	1.000	1.000	0.972	0.709
*OW*	8.50 (6.10–11.00)		8.30 (5.90–11.00)		0.966	8.80 (5.50–13.30)		9.30 (5.60–11.90)		0.938	0.966	0.978
**Eo (%)**	2.90 (0.90–15.90)		2.70 (0.80–6.30)		0.256	2.70 (0.60–5.10)		2.45 (0.70–5.00)		0.987	0.154	0.583
*NW*	3.00 (1.20–8.00)	0.998	2.95 (1.40–5.70)	0.988	1.000	2.60 (1.10–5.10)	1.000	2.30 (0.80–5.00)	1.000	1.000	0.865	0.854
*OW*	2.50 (0.90–15.90)		2.70 (0.80–6.30)		0.196	2.70 (0.60–5.00)		2.50 (0.70–4.50)		1.000	0.712	1.000
**Ba (%)**	0.70 (0.30–1.60)		0.70 (0.20–1.50)		0.346	0.70 (0.30–1.30)		0.60 (0.20–1.10)		0.113	0.621	0.381
*NW*	0.55 (0.30–1.30)	**0.050**	0.45 (0.20–1.10)	**0.028**	0.756	0.70 (0.40–1.30)	0.986	0.60 (0.20–0.80)	0.812	**0.021**	0.793	1.000
*OW*	0.80 (0.40–1.60)		0.80 (0.40–1.50)		0.853	0.70 (0.30–1.10)		0.60 (0.30–1.10)		1.000	0.166	0.454
**Neu (×10^9^/L)**	2.92 (1.58–4.55)		3.04 (1.74–5.29)		0.975	2.84 (1.80–5.01)		2.86 (1.69–7.06)		0.839	0.936	0.981
*NW*	3.02 (2.24–4.16)	0.998	3.14 (1.74–4.64)	1.000	0.988	2.37 (1.89–5.01)	0.820	2.45 (1.75–4.13)	0.341	0.857	0.989	0.931
*OW*	2.92 (1.58–4.55)		2.88 (2.12–5.29)		0.953	3.39 (1.80–4.89)		3.07 (1.69–7.06)		1.000	0.889	0.984
**Ly (×10^9^/L)**	1.93 (0.98–3.05)		2.22 (1.24–3.28)		**0.025**	1.82 (0.83–3.32)		2.06 (1.11–3.17)		0.367	0.986	0.654
*NW*	2.34 (1.17–3.05)	0.412	2.17 (1.24–3.28)	0.968	0.933	1.78 (0.83–3.32)	0.937	2.07 (1.11–2.91)	0.878	0.921	0.572	0.576
*OW*	1.90 (0.98–2.92)		2.24 (1.25–3.13)		**0.030**	2.05 (1.16–2.95)		2.19 (1.27–3.17)		0.745	0.852	1.000
**Mo (×10^9^/L)**	0.48 (0.34–0.67)		0.48 (0.27–0.76)		0.903	0.48 (0.32–0.92)		0.45 (0.30–0.84)		0.910	0.733	0.937
*NW*	0.51 (0.40–0.61)	0.929	0.47 (0.30–0.76)	1.000	0.897	0.47 (0.33–0.69)	0.835	0.44 (0.31–0.84)	0.770	0.993	0.990	0.999
*OW*	0.44 (0.34–0.67		0.50 (0.27–0.76)		0.665	0.51 (0.32–0.92)		0.49 (0.30–0.78)		1.000	0.588	0.952
**Eo (×10^9^/L)**	0.15 (0.07–1.06)		0.16 (0.06–0.33)		0.341	0.13 (0.04–0.30)		0.13 (0.03–0.34)		0.994	0.114	0.503
*NW*	0.19 (0.07–0.48)	1.000	0.21 (0.08–0.27)	0.989	0.998	0.13 (0.06–0.27)	0.986	0.11 (0.03–0.23)	0.971	1.000	0.582	0.642
*OW*	0.14 (0.07–1.06)		0.15 (0.06–0.33		0.448	0.15 (0.04–0.30)		0.15 (0.05–0.34)		1.000	0.828	1.000
**Ba (×10^9^/L)**	0.04 (0.02–0.10)		0.04 (0.01–0.09)		0.740	0.04 (0.02–0.07)		0.04 (0.01–0.07)		0.374	0.507	0.306

*White blood cell counts in the entire study cohort and for the sub-cohorts stratified based on BMI (BMI < 25 kg/m^2^: NW; BMI > 25 kg/m^2^: OW, as determined at recruitment). Data are expressed as median (range) since the non-parametric distribution, as assayed by D’Agostino-Pearson’s test. Within-group (EXP and CTRL) time-dependent changes in NW and OW subjects were performed by the means of two-way ANOVA with Sidak’s multiple comparison post hoc test. Statistically significant (p-values < 0.05) differences are indicated in bold. NW, normal weight subjects; OW, overweight subjects; WBC, white blood cell count; Neu, neutrophils count; Mo, monocytes count; Ly, lymphocytes count; Eo, eosinophils count; Ba, basophils count.*

The two cohorts, compared before and after the intervention (T2-pre for EXP), resulted homogenous for age and most of the measured parameters. Within each cohort, NW and OW subjects differed for weight and BMI at T1 and T2 in both EXP and CTRL. Basophils were increased in OW subjects at both T1 and T2 of the CTRL cohort, while they were reduced between T1 and T2 in the EXP-NW cohort. No time-dependent change was observed in CTRL, although lymphocyte absolute count increased from T1 to T2 in the entire group and in the OW cohort. The results of the ANOVA tests and the related effect size are reported in Supplementary Table 2.

Considering the metabolic markers, HDL was lower in EXP-OW compared to EXP-NW at both time-points, and decreased in NW subject at the end of the training (Supplementary Table 1). In the EXP group, a time-dependent, although clinically irrelevant, reduction of serum iron was recorded in OW subjects, and at T2 compared to CTRL considering the whole population or the OW subgroup. Importantly, baseline 25-(OH)D concentrations did not differ among any of the cohorts.

### Expression and Stability of PPIB, PGK1, and ACTB Genes

**FIGURE 1:**
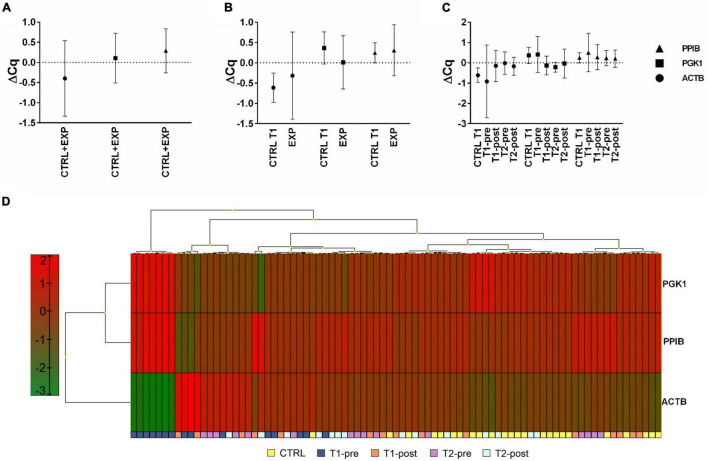
Expression profile of potential housekeeping genes, ACTB, PGK1, and PPIB, in whole blood. Expression profile of the three selected potential reference genes (ACTB, PGK1, and PPIB). Expression level of each potential reference gene was analyzed considering **(A)** samples from all groups (CTRL+EXP), **(B)** samples from the EXP (T1-pre, T1-post, T2-pre, and T2-post), and CTRL groups separated, and **(C)** samples from each time-point separated. **(D)** Heat-map of the expression profiles of ACTB, PGK1, and PPIB, from each group (CTRL, T1-pre, T1-post, T2-pre, and T2-post). Different colors represent differential gene expression.

According to previously published studies (Dheda et al., 2004; Falkenberg et al., 2011), we selected PPIB, PGK1, and ACTB as possible candidates reference genes to normalize RT-qPCR data in whole blood samples. Following a cluster analysis to exclude co-regulation, ΔCq analysis revealed a more scattered expression of ACTB compared to PPIB and PGK1 (Figures 1A–C) and a more constant of PPIB and PGK1 in all samples at all time-points (Figure 1D). Descriptive characteristics of the potential reference genes and the relative expression stability analysis are summarized in Supplementary Tables 3, 4.

The expression stability of candidate genes was evaluated by NormFinder and GeNorm algorithms: PPIB and PGK1 were the best ranked genes, by NormFinder, while the combination PPIB-PGK1 was identified by GeNorm. Therefore, PPIB and PGK1 were used as reference genes.

### Effect of Training on NLRP3 and TLR4 Expression in Whole Blood

**FIGURE 2:**
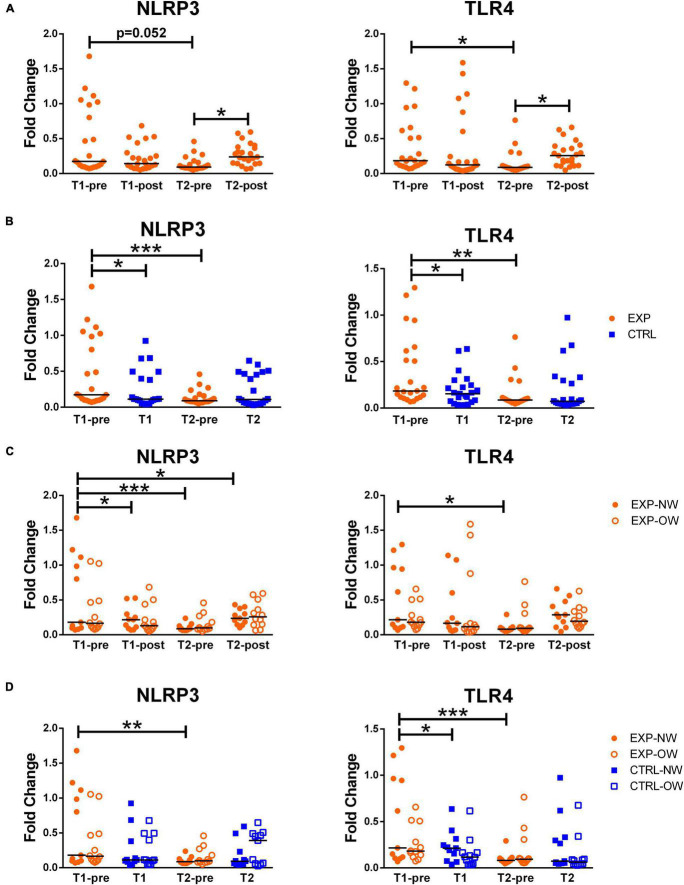
Expression of inflammasome-related genes in whole blood samples. Fold change of NLRP3 and TLR4 gene expression, normalized on PPIB and PGK1, in whole blood from elderly women underwent a 12-week aerobic Nordic walking training program (EXP, *n* = 29) and untrained controls (CTRL, *n* = 35). **(A)** Expression of NLRP3 and TLR4 in the whole EXP group before and after the first (T1-pre and T1-post) and the last (T2-pre and T2-post) sessions of Nordic walking. **(B)** Expression of NLRP3 and TLR4 in the whole EXP group (orange dots), before the first and the last (T1-pre and T2-pre) Nordic walking session, and in the CTRL (blue squares) over the same observation period (T1, T2). **(C)** Expression of NLRP3 and TLR4 in the whole EXP cohort, grouped based on BMI in normal weight (NW, full dots) and overweight (OW, empty dots) subjects. **(D)** Expression of NLRP3 and TLR4 in the EXP (orange dots) and CTRL (blue squares) cohorts, grouped based on BMI in normal weight (NW, full symbols) and overweight (OW, empty symbols) subjects. Asterisks indicated significant differences according to the different statistic tests applied: *p < 0.05; **p < 0.01; ***p < 0.001.

NLRP3 and TLR4 mRNA expression levels were determined in whole blood. Both NLRP3 and TLR4 showed similar expression profiles: while a single bout of aerobic exercise at the beginning of the training protocol (T1-pre vs. T1-post) did not induce any significant changes in the expression of both genes, they were induced at T2-post, compared to T2-pre [NLRP3 T2-pre: 0.090 (0.049–0.459) vs. NLRP3 T2-post: 0.237 (0.064–0.594); TLR4 T2-pre: 0.087 (0.046–0.765) vs. TLR4 T2-post: 0.256 (0.047–0.660)]. Additionally, both NLRP3 and TLR4 expression levels were significantly reduced between T1-pre and T2-pre [NLRP3: 0.172 (0.072–1.680) vs. 0.090 (0.049–0.459); TLR4: 0.184 (0.068–1.296) vs. 0.087 (0.046–0.765)] (Figure 2A).

However, when comparing EXP and CTRL, the two groups differed at the beginning of the training (T1-pre vs. T1) for both genes. While in the CTRL group, no time effect was recorded, the EXP group showed a significant reduction of both genes at T2-pre compared to T1-pre (Figure 2B).

When analyzing the trained cohort sub-grouped in NW and OW, no differences were found at any time-point between the two sub-groups (Figure 2C).

The NW sub-population was affected by the acute intervention since it showed a significant increase in the NLRP3 gene expression [T1-pre vs. T1-post) (0.180 (0.072–1.679) vs. 0.218 (0.069–0.528), respectively] and this acute effect was seen also at the end of the training, when comparing T2-pre vs. T2-post, even though not significantly (Figure 2C). The chronic exercise, on the other hand, induced a significant decrease in both NLRP3 and TLR4 expression at the rest time-points (T1-pre vs. T2-pre) [NLRP3: 0.180 (0.072–1.679) vs. 0.088 (0.062–0.237); TLR4 0.216 (0.068–1.300) vs. 0.081 (0.046–0.292)] in the NW cohort (Figures 2C,D).

When comparing the NW and OW cohorts between the EXP and CTRL groups, besides the lack of differences in NLRP3, the expression level of TLR4 resulted slightly higher, but significant, in EXP-NW than CTRL-NW at the beginning of the training (Figures 2C,D).

The results of the ANOVA tests and the related effect size are reported in Supplementary Table 5.

### Effect of Training on the Release of Inflammasome- and Metabolic Inflammation-Related Cytokines

**FIGURE 3:**
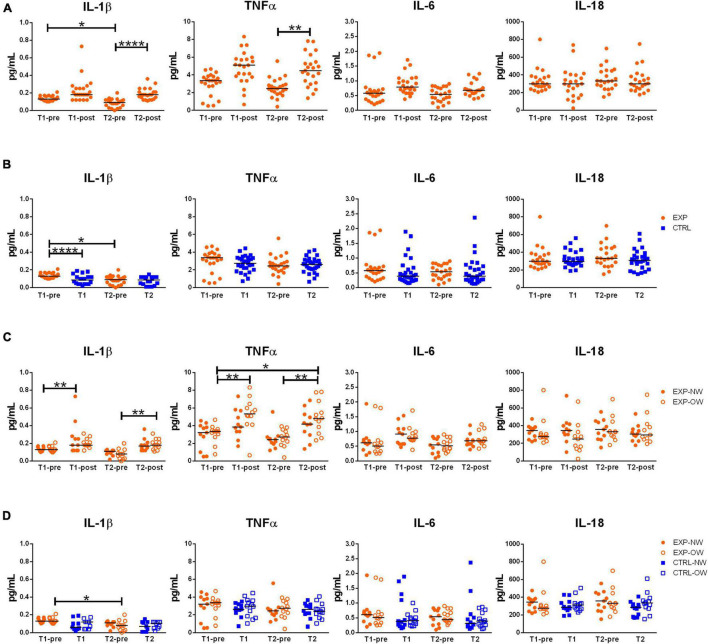
Circulating levels of relevant cytokines associated to inflammasome pathway activation. Concentrations of IL-1β, TNFα, IL-6 and IL-18 in sera from elderly women underwent to a 12-week aerobic Nordic walking training program (EXP, *n* = 29) and untrained controls (CTRL, *n* = 35). **(A)** Circulating levels of the assayed cytokines in the whole EXP group before and after the first (T1-pre and T1-post) and the last (T2-pre and T2-post) sessions of Nordic walking. **(B)** Circulating levels of the assayed cytokines in the whole EXP group (orange dots), before the first and the last (T1-pre and T2-pre) Nordic walking session, and in the CTRL (blue squares) over the same observation period (T1, T2). **(C)** Circulating levels of the assayed cytokines in the EXP cohort, grouped based on BMI in normal weight (NW, full dots) and overweight (OW, empty dots) subjects. **(D)** Circulating levels of the assayed cytokines in the EXP (orange dots) and CTRL (blue squares) cohorts, grouped based on BMI in normal weight (NW, full symbols) and overweight (OW, empty symbols) subjects. Asterisks indicated significant differences according to the different statistic tests applied: *p < 0.05; **p < 0.01; ****p < 0.0001.

In order to determine the effect of the aerobic training program on inflammasome activation and inflammatory status, the circulating concentrations of related cytokines (IL-1β and IL-18, as markers of inflammasome activation, and TNFα and IL-6, as markers of metabolic inflammation) were determined. IL-18 levels neither were affected by the intervention in the EXP group, nor were different between EXP and CTRL groups, or between NW and OW cohorts (Figure 3). A single exercise bout elicited a rise in blood concentrations of the other cytokines, although not significant at the beginning of the training, but highly significant for IL-1β and TNFα at the end of the training (T2-pre vs. T2-post) [IL-1β: 0.090 pg/mL (0.000–0.200) pg/mL vs. 0.180 pg/mL (0.110–0.360) pg/mL; TNFα: 2.450 pg/mL (0.410–5.560) pg/mL vs. 4.490 pg/mL (1.380–7.820) pg/mL], while for IL-6, this increase is not significant (Figure 3A). No changes were detected between the CTRL and the EXP groups at the two time-points except for IL-1β that decreased at the beginning of the training (T1-pre vs. T1, Figure 3B).

When considering the EXP group divided between NW and OW sub-cohorts (Figure 3C), a single bout of exercise induced a general increase of IL-1β in both sub-cohorts, however, significant at the beginning of the training (T1-pre vs. T1-post) for NW [0.130 pg/mL (0.100–0.170) pg/mL vs. 0.180 pg/mL (0.120–0.730) pg/mL] and at the end of the training (T2-pre vs. T2-post) for OW [0.080 pg/mL (0.000–0.200) pg/mL vs. 0.180 pg/mL (0.110–0.310) pg/mL]. On the other hand, as effect of the chronic intervention (T1-pre vs. T2-pre), the cytokine decreased, though not significantly, in both sub-cohorts [NW: 0.130 pg/mL (0.100–0.170) pg/mL vs. 0.110 pg/mL (0.020–0.130) pg/mL; OW: 0.130 pg/mL (0.100–0.210) pg/mL vs. (0.080 pg/mL (0.000–0.200) pg/mL]. For TNFα, only the OW subjects were affected by a modulation of the cytokine, characterized by a strong increase after the first bout compared to baseline (T1-pre vs. T1-post) [3.360 pg/mL (0.770–4.670) pg/mL vs. 5.340 pg/mL (0.660–8.330) pg/mL], a decrease after the 12-week training (T1-pre vs. T2-pre) [2.740 pg/mL (0.410–3.930) pg/mL], and an increase after the last bout of exercise (T2-pre vs. T2-post) [4.830 pg/mL (2.350–7.820) pg/mL]. Additionally, an overall increase of TNFα was observed between the beginning (T1-pre) and the end of the training (T2-post). Even though IL-6 followed the same pattern of the two other cytokines, no differences were significant. Curiously, no differences were observed between the NW and OW cohorts (Figure 3C).

Finally, no differences were observed between EXP and CTRL groups within the stratification in NW and OW (Figure 3D).

The results of the ANOVA tests and the related effect size are reported in Supplementary Table 5.

## Discussion

Our study aimed at analyzing the behavior of inflammasome activation in post-menopausal women performing 12-week moderate-intensity aerobic Nordic walking training. Obtained findings reveal that Nordic walking training program is associated with a reduced expression of inflammasome components at rest and, importantly, the acquisition of a post-exercise pro-inflammatory response at the end of the training period, as indicated by the modulation of NLRP3 and TLR4 mRNA, and IL-1β and TNFα levels. Interestingly, when participants are grouped based on BMI it emerges that NLRP3 response is more pronounced in NW subjects than in OW. The downstream markers of inflammasome activation status, IL-1β, and of the inflammatory status, TNFα, showed a similar course: their level increases after the first bout of exercise, decreases after the 12-week training, and newly increases in response to the last exercise session. Noteworthy, in case of IL-1β, this modulation is significant for NW at the beginning of the training, whereas for OW at the end of the training; in case of TNFα, this time-dependent modulation was significant only in OW. This suggests a beneficial effect of Nordic walking in both NW and OW due to the reduction of inflammasome marker IL-1β. Importantly, vitamin D status, which is known to potentially affect the innate immunity response (Arababadi et al., 2018), was comparable among the EXP and CTRL groups and the relative sub-cohorts.

The importance of studying inflammasome resides in the fact that its activation may be the cause of (or may contribute to) the onset and development of several diseases (Guo et al., 2015) and particularly, since the involvement of NLRP3 inflammasome in age-associated chronic LGI, linked to the onset of several age-related diseases (Mejias-Pena et al., 2017). Aging may represent a key determinant of the responsivity to aerobic exercise. In a recent study, an 8-week Nordic walking training reduced the TLR4 and NLRP3 mRNA expression and circulating levels of IL-1β and IL-18 in young males (Khakroo Abkenar et al., 2019) while, in the present study, the most relevant result, is represented by the (re)acquisition of a post-acute exercise inflammatory response. In order to investigate whether the activation status of the inflammasome machinery, in response to aerobic activity in elderly women, was reflected into the circulation, mRNA expression level of NLRP3 and TLR4 was analyzed in whole blood. The choice of whole blood, as the assay matrix, was driven by the fact that inflammasome pathways can be activated in virtually all blood cells, but red blood cells (Tran et al., 2019), other than in other tissues. Therefore, whole blood expression of inflammasome markers may picture the integrated innate response potential against danger signals which may drive, in turn, LGI (Gomarasca et al., 2020). Gene expression analysis is completed by a normalization study to determine the best suitable reference genes in RT-qPCR analysis, in order to obtain the most reliable results (Mahoney et al., 2004; Faraldi et al., 2018).

Exercise training-dependent alteration of immune function is associated with the activation of several local and systemic responses. For instance, exercise activates purinergic signaling (ATP, ADP, adenosine, related receptors, and enzymes) (Moritz et al., 2021). The purinergic system has relevance in inflammatory response and, particularly, in the shift from the pro-inflammatory response to acute, intense exercise to the anti-inflammatory response associated with chronic exercise (Cardoso et al., 2021). Exercise improves immune functions via the stimulation of the neuroendocrine secretion of catecholamines and the activation of their signaling (Simpson et al., 2021). Similarly, PA, together with diet, affects the composition of gut microbiota with profound effects on immune function, but also on muscle strength and dynamics (Strasser et al., 2021).

Many pieces of evidence have demonstrated that PA has inhibitory effects on inflammasome activation. This control may take place in different ways, indirectly by targeting pro-inflammatory compounds (fFA, ceramides) that are increased in LGI and aging (Ringseis et al., 2015) or, as it happens consequently to neuronal stimulation of myofibers, via the perturbation of plasma membrane integrity and potassium gradient across the membrane (Gaidt and Hornung, 2018), and directly by triggering TLRs expression and their downstream signaling. For instance, IL-18, the designated marker of NLRP3 inflammasome activation status was decreased by 43% in men and women with metabolic syndrome in response to a 12-week aerobic interval training program (three times a week) (Stensvold et al., 2012). Similarly, IL-18 was decreased in patients with metabolic syndrome undergone 12-week combined (endurance and strength) training program (three times a week) (Troseid et al., 2009), in T2DM subjects following a 6-month aerobic moderate-intensity exercise training program (four times/week) (Kadoglou et al., 2007a,b), and 8-week high-intensity exercise training on a rowing ergometer (three times/week), in obese subjects (Leick et al., 2007). However, similarly to our results, other authors failed in evidencing any putative improvement of the inflammatory status marked by IL-18. For instance, Christiansen et al. (2010) did not observe any decrease in circulating IL-18 concentrations in obese men and women following a 12-week aerobic exercise training program performed three times a week, possibly because of the relatively moderate intensity of the exercise. Also, RT has been shown to target innate immunity and inflammasome activation: in healthy elderly 8-week RT decreased the protein expression of TLR2 and TLR4 as well as the expression of several TLRs signaling-associated molecules (e.g., MyD88, TRIF, NF-κB, and MAPK) and plasma levels of the CRP (Rodriguez-Miguelez et al., 2014). On the contrary, Mejias-Pena et al. (2016) did not record any change in the expression of TLR2, TLR4, MyD88, and TRIF, in peripheral blood mononuclear cells (PBMCs) from older subjects after 8 weeks of aerobic exercise training, suggesting the possibility that the type of exercise might be a determinant of the TLRs-mediated anti-inflammatory effect of exercise. The lack of control for confounding variables, in the available studies, prevents a definitive elaboration on potential benefits of this interventional practice, according to a recently published systematic review (Sanchez-Lastra et al., 2020). However, Nordic walking is a potentially beneficial exercise strategy for overweight and obese people. Based on the twelve good-to-fair quality selected studies, the authors of this study evidenced that subjects performing Nordic walking experienced significant improvement in parameters such as fasting plasma glucose, abdominal adiposity, and body fat compared with the values recorded at baseline, but no significant improvements were found when compared with control groups (Sanchez-Lastra et al., 2020). Notably, adiposity in the elderly may be associated with a deregulated glucose metabolism, and hence to an increased risk of T2DM, via a deregulation of immune cells and, specifically of CD8+ cytotoxic subsets (Bosslau et al., 2021).

In regards to the inflammasome activation status, our results revealed that the last bout of exercise determined an increase of both NLRP3 and TLR4 mRNA, indicating that, possibly, the ability to activate inflammasome is acquired during the training program, since such a response was absent after only one bout of exercise at the beginning of the training. This effect may be linked to the restoration of whole blood NLRP3/TLR4 mRNA expression at the end of the program, compared to the first sampling. As a consequence, these results demonstrated that although the circulating inflammatory profile may be fairly affected by the moderate-intensity aerobic training, the system alertness to endogenous and exogenous danger signals (TLR4 and NLRP3 mRNA) may be restored by the activity. Still, few limitations must be mentioned. The CTRL group, in our study, was not involved in any training program and, hence, the comparison was made between Nordic walking and sedentary lifestyle. Therefore, future investigations should aim at comparing the effect of different kinds of physical activities. This study lacks an objective and standardized method of assessment of the participants’ effort, as well as of their baseline physical activity level [e.g., via the international physical activity questionnaire (IPAQ)] and thereby, it is not possible to define if the effects on inflammasome activation and inflammatory response may be related to the effort spent. Only females have been considered and for future research gender-dependent differences should also be considered. Other limitations are related to the eventual lack of comparison between subjects with different inflammatory statuses at the beginning of the training. This additional comparison would highlight the effect of Nordic Walking training on inflammation. Further, gene expression analysis of cytokines would have given additional information related to the inflammatory status of PBMCs while their circulating levels represent the net result of their systemic expression. Finally, body composition analysis would have a greater significance than anthropometrical measures in describing the cohorts and, eventually, their intervention-related changes. An important strength of this study is represented by the robust study of normalization, based on validated algorithms, applied to gene expression analysis: indeed, different normalization strategies may lead to different results and, for each experimental set, it is recommended to select the most appropriate method.

## Conclusion

**FIGURE 4:**
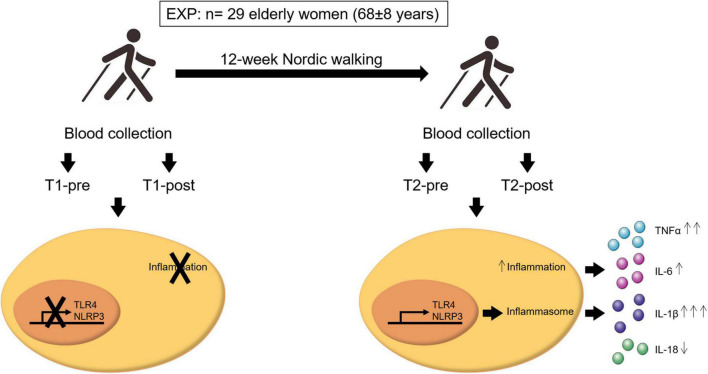
Schematic representation of the important findings of this study. A population of 29 elderly women (age 68 ± 8 years old) completed 12 weeks of Nordic walking moderate-intensity aerobic training program (EXP). Blood samples were taken before and after the first (T1-pre and T1-post, respectively) and last (T2-pre and T2-post, respectively) exercise unit, and inflammasome- and inflammation-related markers were assessed. Aerobic training mildly affected the inflammasome-related inflammatory response. Participants acquired an acute inflammatory response to exercise, that was absent at baseline, that resulted in the activation of NLRP3 and TLR4 gene expression and the consequent release in the blood stream of the inflammasome- and inflammation-related cytokines IL-1β, IL-6, TNFα, and IL-18. In particular, a single bout of exercise at the end of the training induced a significant upregulation of IL-1β (↑↑↑) and TNFα (↑↑), a non-significant increase of IL-6 (↑), and a non-significant down-modulation of IL-18 (↓). The arrows next to the cytokines refer to the entity of the effect of the upregulation or downregulation of these cytokines.

Despite a fair effect on the resting whole blood expression of inflammasome constituents (NLRP3 and TLR4) and circulating levels of the downstream inflammasome related cytokine IL-1β, a 12-week moderate-intensity aerobic training program (Nordic walking) allows the partial acquisition of the acute exercise-induced inflammatory response at the end of the training compared to the total absence of response observed at the beginning of the program (Figure 4). Specifically, a post-training acute exercise-induced response was recorded for NLRP3 and TLR4 expression, while IL-1β and TNFα changes were driven by the overweight participants.

## Data Availability Statement

The datasets presented in this study can be found in online repositories. The names of the repository/repositories and accession number(s) can be found below: https://zenodo.org/, 10.5281/zenodo.5789001.

## Ethics Statement

The studies involving human participants were reviewed and approved by the Bioethical Committee of the Regional Medical Society in Gdansk. The patients/participants provided their written informed consent to participate in this study.

## Author Contributions

EZ and GL: conception. MG, KM, EZ, and GL: study design. KM and MFl: data acquisition. MG, KM, MFa, MFl, and SP: data analysis. MG, KM, MFa, EZ, and GL: data interpretation. MG, KM, MFa, and GL: manuscript drafting. MFl, SP, GB, and EZ: manuscript revision. MG, KM, MFa, MFl, SP, GB, EZ, and GL: final approval. All authors contributed to the article and approved the submitted version.

## Conflict of Interest

The authors declare that the research was conducted in the absence of any commercial or financial relationships that could be construed as a potential conflict of interest.

## Publisher’s Note

All claims expressed in this article are solely those of the authors and do not necessarily represent those of their affiliated organizations, or those of the publisher, the editors and the reviewers. Any product that may be evaluated in this article, or claim that may be made by its manufacturer, is not guaranteed or endorsed by the publisher.
