# Supplementary material for: Impact of 12-Week Moderate-Intensity Aerobic Training on Inflammasome Complex Activation in Elderly Women
Source: Front Physiol. 2022 Feb 22;13:792859. doi: 10.3389/fphys.2022.792859 (PMC8902397; doi:10.3389/fphys.2022.792859)
Supplement: Supplementary file 1 [file Table_1.PDF]

Supplementary Table 1. Characterization of the metabolic markers of the study cohort.

	CTRL (n=29)					EXP (n= 29)						
	T1		T2		p-value T1 vs. T2	T1-pre		T2-pre		p-value T1-pre vs. T2-pre	p-value T1 CTRL vs. T1-pre EXP	p-value T2 CTRL vs. T2-pre EXP
	Median (min-to-max)	p-value NW vs. OW	Median (min-to-max)	p-value NW vs. OW		Median (min-to-max)	p-value NW vs. OW	Median (min-to-max)	p-value NW vs. OW			
**TChol (mg/dL)**	232.0 (155.0-434.0)		213.0 (144.0-491.0)		0.393	232.5 (152.0-275.0)		223.0 (162.0-304.0)		0.684	0.998	0.954
NW	251.50 (157.0-434.0)	**0.047**	218.50 (173.0-491.0)	0.124	0.681	234.0 (167.0-275.0)	1.000	223.0 (177.0-284.0)	1.000	0.928	0.735	0.888
OW	210.0 (155.0-276.0)		204.0 (144.0-276.0)		0.957	225.0 (152.0-264.0)		216.0 (162.0-304.0)		0.992	0.868	0.814
**HDL (mg/dL)**	69.0 (44.0-103.0)		67.0 (46.0-95.0)		0.502	68.50 (51.0-157.0)		65.50 (50.0-115.0)		0.101	0.818	0.964
NW	78.0 (56.0-99.0)	0.506	78.0 (57.0-92.0)	0.623	0.837	78.0 (58.0-157.0)	**<0.001**	77.0 (58.0-115.0)	**0.008**	**0.040**	0.455	0.882
OW	63.0 (44.0-103.0)		61.0 (46.0-95.0)		0.951	60.0 (51.0-90.0)		60.0 (50.0-95.0)		0.998	0.932	0.967
**LDL (mg/dL)**	132.0 (70.0-356.0)		114.0 (50.0-393.0)		0.367	130.50 (69.0-187.0)		124.0 (72.0-221.0)		0.806	0.992	0.976
NW	135.0 (70.0-356.0)	0.197	114.0 (79.0-393.0)	0.359	0.683	119.0 (69.0-183.0)	0.954	113.0 (81.0-193.0)	0.983	0.999	0.458	0.782
OW	131.0 (71.0-187.0)		114.0 (50.0-189.0)		0.940	134.0 (74.0-187.0)		127.0 (72.0-221.0)		0.965	0.751	0.734
**TG (mg/dL)**	104.0 (69.0-279.0)		117.0 (58.0-216.0)		0.866	107.0 (53.0-201.0)		109.50 (66.0-198.0)		0.389	0.749	0.989
NW	102.50 (69.0-163.0)	0.979	108.0 (58.0-207.0)	0.996	0.986	85.0 (53.0-149.0)	0.333	93.0 (68.0-163.0)	0.307	0.895	0.898	0.970
OW	104.0 (85.0-279.0)		117.0 (66.0-216.0)		0.998	123.0 (79.0-201.0)		129.0 (66.0-198.0)		0.829	1.000	0.990
**Iron (µg/dL)**	101.0 (4.0-167.0)		106.0 (60.0-156.0)		0.118	97.0 (49.0-150.0)		85.50 (51.0-154.0)		0.073	0.980	**0.006**
NW	101.0 (4.0-142.0)	0.935	102.0 (60.0-156.0)	0.989	0.499	88.0 (49.0-133.0)	0.537	91.0 (60.0-154.0)	1.000	0.998	1.000	0.552
OW	104.0 (59.0-167.0)		106.0 (75.0-154.0)		0.579	110.0 (65.0-150.0)		85.0 (51.0-120.0)		**0.039**	0.997	**0.049**
**Ferritin (ng/dL)**	113.80 (28.0-379.40)		106.80 (29.10-294.30)		0.064	94.85 (31.0-378.50)		90.30 (31.70-491.10)		0.555	0.794	0.990
NW	94.35 (28.0-288.30)	0.727	90.95 (36.30-251.80)	0.622	0.316	79.50 (31.0-378.50)	0.999	86.60 (31.70-491.10)	1.000	0.773	1.000	0.971

OW	136.60 (34.40-379.40)		160.30 (29.10-294.30)		0.536	115.0 (50.10-331.30)		96.70 (41.30-334.70)		0.995	0.968	0.999
**25-(OH)D (ng/dL)**	32.40 (22.10-53.80)		n.a.		/	31.15 (22.30-43.60)		n.a.		/	0.550	/
NW	36.10 (25.20-48.60)	0.521	n.a	/	/	29.80 (22.30-43.60)	0.582	n.a	/	/	0.108	/
OW	30.85 (22.10-53.80)		n.a		/	32.20 (28.50-40.30)		n.a		/	0.986	/

Description of hematologic markers in the entire study cohort and for the sub-cohorts stratified based on BMI (BMI< 25 kg/m^2: NW; BMI> 25 kg/m^2: OW, as determined at recruitment). Data are expressed as median (range) since the non-parametric distribution, as assayed by the D'Agostino-Pearson's test. Comparison of 25-OH Vitamin D between EXP and CTRL were assessed using Mann-Whitney test. Within-group (EXP and CTRL) time-dependent changes in NW and OW subjects was performed by the means of 2-way ANOVA with Sidak's multiple comparison post-hoc test. Statistically significant (p-values< 0.05) differences are indicated in bold. Abbreviations: NW: normal weight subjects; OW: overweight subjects; TChol: total cholesterol; HDL: high-density lipoprotein cholesterol; LDL: lOW-density lipoprotein cholesterol; TG: triglyceride; 25-(OH)D: 25-OH Vitamin D; n.a: not available.

Supplementary Table 2. Results of ANOVA measures and calculation of effect size for the anthropometrical characteristic and blood cell counts of the study cohorts. The effect size was calculated by Kendal W for Friedman's tests, Cohen's eta-squared for 2-way ANOVA, and Cohen d for the post-hoc tests. Values are reported for the significant comparisons only.

Comparison	*Source of variation*	χ^2	*DFn*	*DFd*	*Effect Size*	*p-value*
HEIGHT						
**EXP NW/OW vs. CTRL NW/OW (two way ANOVA)**	Interaction	0.379	1	52	0.007	0.541
	Time	1.533	1		0.029	0.221
	Activity	0.986	1		0.019	0.325
AGE						
**EXP NW/OW vs. CTRL NW/OW (two way ANOVA)**	Interaction	1.48	1	53	0.027	0.2295
	Time	0.082	1		0.002	0.7757
	Activity	0.256	1		0.005	0.6151
WEIGHT						
**EXP vs. CTRL (two way ANOVA)**	Interaction	1.091	1	55	0.019	0.301
	Time	0.317	1		0.006	0.576
	Activity	0.576	1		0.010	0.451
**EXP NW/OW vs. CTRL NW/OW (two way ANOVA)**	Interaction	1.124	3	53	0.060	0.348
	Time	0.270	1		0.005	0.606
	Activity	10.10	3		0.364	< 0.0001
EXP NW T1 vs. EXP OW T1			106		0.175	< 0.001
CTRL NW T1 vs. CTRL OW T1			106		0.133	0.004
EXP NW T2 vs. EXP OW T2			106		0.161	< 0.001
CTRL NW T2 vs. CTRL OW T2			106		0.265	0.004
BMI						
**EXP vs. CTRL (two way ANOVA)**	Interaction	0.043	1	55	0.001	0.836
	Time	0.115	1		0.002	0.736
	Activity	2.165	1		0.038	0.147
**EXP NW/OW vs. CTRL NW/OW (two way ANOVA)**	Interaction	0.352	3	53	0.020	0.788
	Time	0.070	1		0.001	0.792
	Activity	25.49	3		0.591	< 0.0001
EXP NW T1 vs. EXP OW T1			104		0.267	< 0.0001
CTRL NW T1 vs. CTRL OW T1			104		0.230	< 0.0001
EXP NW T2 vs. EXP OW T2			104		0.242	< 0.0001
CTRL NW T2 vs. CTRL OW T2			104		0.203	< 0.0001
WBC						
**EXP vs. CTRL (two way ANOVA)**	Interaction	0.604	1	55	0.011	0.441
	Time	0.588	1		0.011	0.446
	Activity	0.073	1		0.001	0.788
**EXP NW/OW vs. CTRL NW/OW (two way ANOVA)**	Interaction	0.881	3	53	0.048	0.457
	Time	0.331	1		0.006	0.568
	Activity	1.412	3		0.074	0.250
NEU%						
**EXP vs. CTRL (two way ANOVA)**	Interaction	0.140	1	55	0.003	0.710
	Time	2.481	1		0.043	0.121
	Activity	0.061	1		0.001	0.807
**EXP NW/OW vs. CTRL NW/OW (two way ANOVA)**	Interaction	0.073	3	53	0.004	0.974
	Time	2.462	1		0.044	0.123
	Activity	0.346	3		0.019	0.792
LY%						
**EXP vs. CTRL (two way ANOVA)**	Interaction	0.002	1	55	0.00003	0.966
	Time	4.137	1		0.070	0.047
	Activity	0.017	1		0.000	0.898
	Interaction	0.032	3	53	0.002	0.992

**EXP NW/OW vs. CTRL NW/OW (two way ANOVA)**	Time	4.000	1		0.070	0.051
	Activity	0.456	3		0.025	0.714
		MO%				
**EXP vs. CTRL (two way ANOVA)**	Interaction	0.264	1	55	0.005	0.609
	Time	1.281	1		0.023	0.263
	Activity	1.841	1		0.032	0.180
**EXP NW/OW vs. CTRL NW/OW (two way ANOVA)**	Interaction	0.262	3	53	0.015	0.853
	Time	1.299	1		0.024	0.260
	Activity	0.622	3		0.034	0.604
		EO%				
**EXP vs. CTRL (two way ANOVA)**	Interaction	0.909	1	55	0.016	0.345
	Time	1.346	1		0.024	0.251
	Activity	2.248	1		0.039	0.140
**EXP NW/OW vs. CTRL NW/OW (two way ANOVA)**	Interaction	0.858	3	53	0.046	0.469
	Time	0.970	1		0.018	0.329
	Activity	0.769	3		0.042	0.517
		BA%				
**EXP vs. CTRL (two way ANOVA)**	Interaction	0.206	1	55	0.004	0.652
	Time	5.328	1		0.088	0.025
	Activity	1.369	1		0.024	0.247
**EXP NW/OW vs. CTRL NW/OW (two way ANOVA)**	Interaction	1.600	3	53	0.083	0.201
	Time	6.140	1		0.104	0.016
	Activity	3.717	3		0.174	0.017
EXP NW T1 vs. EXP NW T2			53		0.827	0.021
CTRL NW T1 vs. CTRL OW T1			106		0.747	0.050
CTRL NW T2vs. CTRL OW T2			106		0.890	0.028
		NEU				
**EXP vs. CTRL (two way ANOVA)**	Interaction	0.270	1	55	0.005	0.606
	Time	0.055	1		0.001	0.815
	Activity	0.007	1		0.000	0.934
**EXP NW/OW vs. CTRL NW/OW (two way ANOVA)**	Interaction	0.436	3	53	0.024	0.728
	Time	0.127	1		0.002	0.723
	Activity	0.999	3		0.053	0.401
		LY				
**EXP vs. CTRL (two way ANOVA)**	Interaction	0.802	1	55	0.014	0.375
	Time	7.437	1		0.119	0.009
	Activity	0.276	1		0.005	0.602
CTRL T1 vs. CTRL T2			55		0.040	0.025
**EXP NW/OW vs. CTRL NW/OW (two way ANOVA)**	Interaction	0.807	3	53	0.044	0.495
	Time	6.406	1		0.108	0.014
	Activity	1.086	3		0.058	0.363
CTRL OW T1 vs. CTRL OW T2			53		0.063	0.030
		MO				
**EXP vs. CTRL (two way ANOVA)**	Interaction	0.313	1	55	0.006	0.578
	Time	0.000	1		0.000	0.996
	Activity	0.298	1		0.005	0.588
**EXP NW/OW vs. CTRL NW/OW (two way ANOVA)**	Interaction	0.739	3	53	0.040	0.534
	Time	0.029	1		0.001	0.866
	Activity	0.711	3		0.039	0.550
		EO				
**EXP vs. CTRL (two way ANOVA)**	Interaction	0.744	1	55	0.013	0.392
	Time	1.012	1		0.018	0.319
	Activity	2.911	1		0.050	0.094
	Interaction	0.446	3	53	0.025	0.721

**EXP NW/OW vs. CTRL NW/OW (two way ANOVA)**	Time	0.802	1		0.015	0.375
	Activity	1.199	3		0.064	0.319
BA						
**EXP vs. CTRL (two way ANOVA)**	Interaction	0.177	1	55	0.003	0.676
	Time	1.945	1		0.034	0.169
	Activity	1.787	1		0.031	0.187
**EXP NW/OW vs. CTRL NW/OW (two way ANOVA)**	Interaction	2.579	3	53	0.127	0.063
	Time	3.048	1		0.054	0.087
	Activity	3.120	3		0.150	0.034
EXP NW T1 vs. EXP NW T2			53		2.845	0.046
CTRL NW T2 vs. CTRL OW T2			106		0.877	0.025
Vitamin D						
**EXP NW/OW vs. CTRL NW/OW (two way ANOVA)**	Interaction	1.940	1	50	0.037	0.170
	Time	0.012	1		0.0002	0.913
	Activity	2.530	1		0.048	0.118

Supplementary Table 3. List and characteristics of the potential reference genes.

**Gene**	**Acronym**	**Function**	**Chromosome localization**
β-actin	ACTB	Structural role in cytoskeleton	7p22.1
phosphoglycerate kinase	PGK1	glycolysis	Xq21.1
peptidylprolyl isomerase B	PPIB	Association with secretory pathways Cyclosporine-binding protein Regulation of cyclosporine A-mediated immunosuppression. Variants associated with recessive forms of osteogenesis imperfecta.	15q22.31

Source: National Center for Biotechnology Information (https://www.ncbi.nlm.nih.gov/home/genes/)

Supplementary Table 4. Expression stability analysis of ACTB, PGK1, and PPIB.

GROUP	GENE	GeNorm (M)	NormFinder (Stability Value)
CTRL + EXP	PPIB	0.695	0.353
	PGK1		0.599
	ACTB	1.185	1.362
EXPs	PPIB	0.713	0.444
	PGK1		0.557
	ACTB	1.318	1.567
CTRL	PPIB	0.475	0.102
	ACTB		0.464
	PGK1	0.58	0.546
T1-pre	PGK1	0.399	0.199
	PPIB		0.478
	ACTB	1.862	2.681
T1-post	PGK1	0.776	0.205
	PPIB		0.748
	ACTB	1.059	1.084
T2-pre	PGK1	0.308	0.154
	PPIB		0.424
	ACTB	0.651	0.816
T2-post	PPIB	0.485	0.325
	ACTB		0.359
	PGK1	0.885	1.046

Expression stability analysis was performed trough NormFinder and GeNorm algorithm provided by the GenEx software. Expression stability of each potential reference genes was analyzed considering samples of all groups (CTRL+EXP), for samples in the EXP (T1-pre, T1-post, T2-pre, and T2-post), and CTRL groups separated, and for samples in each time-point separated. Stability value, calculated by NormFinder and M value, calculated by GeNorm are reported for each analysis.

Supplementary Table 5. Results of ANOVA measures and calculation of effect size for the inflammasome- or inflammation-related markers. The effect size was calculated by Kendal W for Friedman's tests, Cohen's eta-squared for 2-way ANOVA, and Cohen d for the post-hoc tests. Values are reported for the significant comparisons only.

Comparison	*Source of variation*	χ^2	*DFn*	*DFd*	*Effect Size*	*p-value*
			NLRP3			
**EXP T2 vs. EXP T1 (Friedman Test)**		11.090	3		1.447	0.011
T2-pre vs. T1-pre					0.415	0.052
T2-post vs. T2-pre					0.198	0.012
**EXP vs. CTRL (two way ANOVA)**	Interaction	7.880	1	43	0.155	0.008
	Time	8.920	1		0.172	0.005
	Activity	0.674	1		0.015	0.416
EXP T1 vs. EXP T2			43		0.934	0.000
CTRL T1 vs. EXP T1			86		0.557	0.037
**EXP NW vs. EXP OW (two way ANOVA)**	Interaction	1.560	3	63	0.069	0.207
	Time	6.360	3		0.232	0.001
	BMI	0.421	1		0.007	0.524
T1-pre NW vs. T1-post NW			63		0.829	0.015
T1-pre NW vs. T2-pre NW			63		0.863	0.000
T1-pre NW vs. T2-post NW			63		0.785	0.028
**EXP NW/OW vs. CTRL NW/OW (two way ANOVA)**	Interaction	1.640	3	82	0.057	0.187
	Time	5.040	1		0.058	0.003
	Activity	0.079	3		0.003	0.779
EXP T1-pre NW vs. EXP T2-pre NW			82		0.863	0.002
			TLR4			
**EXP T2 vs. EXP T1 (Friedman Test)**		13.240	3		1.727	0.004
T2-pre vs. T1-pre					0.829	0.010
T2-post vs. T2-pre					0.798	0.010
**EXP vs. CTRL (two way ANOVA)**	Interaction	5.950	1	44	0.119	0.019
	Time	5.930	1		0.119	0.019
	Activity	1.670	1		0.037	0.203
EXP T1 vs. EXP T2			44		0.830	0.003
CTRL T1 vs. EXP T1			88		0.662	0.023
**EXP NW vs. EXP OW (two way ANOVA)**	Interaction	1.460	3	63	0.065	0.234
	Time	3.010	3		0.125	0.037
	BMI	0.341	1		0.005	0.566
T1-pre NW vs. T2-pre NW			63		0.785	0.013
**EXP NW/OW vs. CTRL NW/OW (two way ANOVA)**	Interaction	1.910	3	84	0.064	0.134
	Time	4.530	3		0.139	0.005
	Activity	3.340	1		0.038	0.071
EXP T1-pre NW vs. CTRL T1 NW			84		0.798	0.043
EXP T1-pre NW vs. EXP T2-pre NW			84		0.784	0.001
			IL-1β			
**EXP T2 vs. EXP T1 (Friedman Test)**		33.220	3		4.333	< 0.0001
T2-pre vs. T1-pre					1.874	0.037
T2-post vs. T2-pre					1.220	< 0.0001
**EXP vs. CTRL (two way ANOVA)**	Interaction	4.910	1	39	0.112	0.033
	Time	15.900	1		0.290	0.000
	Activity	4.880	1		0.111	0.033
EXP T1 vs. EXP T2			39		1.873	< 0.0001
CTRL T1 vs. EXP T1			78		1.766	0.006
	Interaction	0.714	3	63	0.033	0.547

**EXP NW vs. EXP OW (two way ANOVA)**	Time	14.600	3		0.410	< 0.0001
	BMI	0.662	1		0.010	0.425
T1-pre NW vs. T1-post NW			63		0.538	0.003
T2-pre OW vs. T2-post OW			63		1.017	0.004
**EXP NW/OW vs. CTRL NW/OW (two way ANOVA)**	Interaction	0.536	3	74	0.021	0.659
	Time	6.760	3		0.215	0.000
	Activity	0.056	1		0.001	0.813
EXP T1-pre OW vs. EXP T2-pre OW			74		1.374	0.015
TNFα						
**EXP T2 vs. EXP T1 (Friedman Test)**		20.100	3		2.622	< 0.001
T2-post vs. T2-pre					1.358	0.003
**EXP vs. CTRL (two way ANOVA)**	Interaction	0.495	1	50	0.010	0.485
	Time	2.380	1		0.045	0.129
	Activity	0.481	1		0.010	0.491
**EXP NW vs. EXP OW (two way ANOVA)**	Interaction	0.554	3	63	0.026	0.648
	Time	12.500	3		0.373	< 0.0001
	BMI	3.090	1		0.047	0.093
T1-pre OW vs. T1-post OW			63		1.456	0.003
T1-pre OW vs. T2-post OW			63		1.237	0.022
T2-pre OW vs. T2-post OW			63		1.550	0.002
**EXP NW/OW vs. CTRL NW/OW (two way ANOVA)**	Interaction	0.091	3	88	0.003	0.965
	Time	1.030	3		0.034	0.385
	Activity	0.501	1		0.006	0.481
IL 6						
**EXP T2 vs. EXP T1 (Friedman Test)**		12.700	3		1.657	0.005
**EXP vs. CTRL (two way ANOVA)**	Interaction	0.832	1	50	0.016	0.366
	Time	0.922	1		0.018	0.342
	Activity	0.476	1		0.009	0.493
**EXP NW vs. EXP OW (two way ANOVA)**	Interaction	0.120	3	63	0.006	0.948
	Time	4.570	3		0.179	0.006
	BMI	0.092	1		0.001	0.764
**EXP NW/OW vs. CTRL NW/OW (two way ANOVA)**	Interaction	0.528	3	89	0.017	0.665
	Time	0.670	3		0.022	0.573
	Activity	0.778	1		0.009	0.380
IL 18						
**EXP T2 vs. EXP T1 (Friedman Test)**		2.730	3		0.372	0.436
**EXP vs. CTRL (two way ANOVA)**	Interaction	1.840	1	48	0.037	0.181
	Time	0.776	1		0.016	0.383
	Activity	0.807	1		0.017	0.373
**EXP NW vs. EXP OW (two way ANOVA)**	Interaction	2.590	3	60	0.115	0.061
	Time	1.150	3		0.054	0.337
	BMI	0.052	1		0.001	0.822
**EXP NW/OW vs. CTRL NW/OW (two way ANOVA)**	Interaction	0.398	3	84	0.014	0.755
	Time	1.050	3		0.036	0.373
	Activity	1.190	1		0.014	0.279
